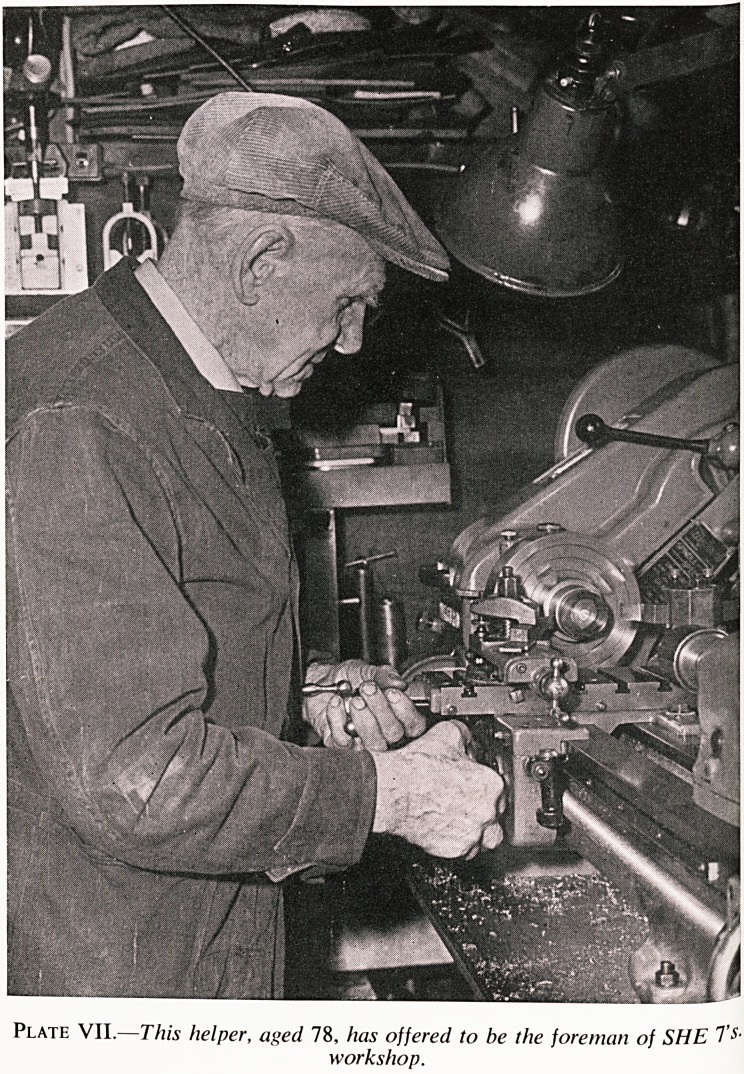# The Care of the Elderly

**Published:** 1969-04

**Authors:** W. H. Lloyd, A. W. Macara, P. S. Sinclair


### THE CARE OF THE ELDERLY.

### I. A GERIATRICIAN'S VIEWPOINT

BY

DR. W. H. LLOYD

The practice of geriatric medicine is complex, involving many different groups of people and many different disciplines. Many people are uncertain as to the function of a geriatric service in the hospital and in the community. Great differences still exist between the part played by different geriatric units in their local hospital services and communities (Boucher 1957). Even now, geriatric units vary from long stay annexes of a type described by Professor A. P. Thomson in 1949, to a very few well equipped and staffed units which are integral members of their hospital group.

In view of this it is not surprising that misunderstanding and resultant conflict are common in this field. Our approach to geriatric medicine is based on the following assumptions :—

(1) Illness, much of it preventable, some of it treatable, all of it open to constructive palliation, is the main enemy of the old.

(2) A process of normal ageing remains to be defined. Meantime there is little evidence that a previously self-sufficient adult ever becomes incapable of caring for himself merely as a result of the passage of time. Senility is no diagnosis. The term is often used as though it were and as an excuse for doing nothing medically for someone in need of medical help. Anyone who describes a person as senile tells more of himself than the person he seeks to describe. The doctor who finds himself faced with an elderly woman incapable of caring for herself should first ask himself why is this old lady as she is and not who will care for her.

(3) Liability to disease and consequent disability increases with age. Chronic illness and disability weaken the will to live as apart from merely continuing to exist. The care of such people should encourage them to live even dangerously. Most of us in our medical training have been taught about the dangers of inducing a cardiac neurosis; how many of us appreciate the long-term danger of inducing an " I'm too old to do anything " neurosis?

(4) Mental inactivity probably, and physical inactivity certainly, are responsible for much crippling and dependence. It is quite terrifying to witness how quickly elderly people become bedridden merely as a result of not walking.

Dorothy Wedderburn (1966) in a recent review paper devoted to the services for elderly people has said " If we try to assess the stage reached by all the varied research which has been going on and which has been concerned with ageing and its problems, it is probably true to say that we have come to the parting of the ways. To put it provocatively, we are moving out of the era of ' mythology ' into an era where at least the problems and the framework in which these problems should be studied can be fairly clearly defined."

Amulree (1968), discussing the relationship of geriatrics to medicine, has pointed out that most doctors engaged in this work appreciate that geriatrics is not a new speciality in medicine, it merely allows normal medical practice to be made available to a large number of ill people to whom it had hitherto been denied. That there is a need to extend medical services for the elderly both within and without the hospital scene can no longer be denied. To achieve this will mean change. This paper outlines the way I envisage geriatric services may develop.

First let me remind you briefly of the extent of disabilities in the elderly population. Sheldon (1948) in his Wolverhampton survey of a random sample of a population of women over sixty and men over sixty-five, found a high incidence of disabilities. Eight per cent were confined to the house and just over twenty per cent had only limited outside movement. Nearly forty per cent had difficulty in managing stairs. Similar findings were reported by Hobson and Pemberton from Sheffield in 1955. Williamson and his colleagues (1966) carried out a full physical and psychiatric examination on a random sample of elderly men and women on the lists of three general practitioners in Scotland. The aim of this study was to discover to what extent the health and social needs of old people were being met. They found that men had a mean of 3.26 disabilities of which 1.87 were not known to the general practitioner, and women had a mean of 3.42 disabilities of which 2.03 were not known. More recently, Exton-Smith and Millard (1968) have carried out a similar study in the London Borough of Camden which has confirmed the work of Williamson. Eighteen per cent of the subjects were anæmic with Hb. levels of less than 11.8 gm/100 ml., eleven per cent had urinary tract infections, four per cent had diabetes and four per cent of the elderly women had osteomalacia. If you apply these figures to the elderly population there is a huge case load.

The findings of Townsend and Wedderburn (1965) in a national survey of elderly people aged sixty-five and over, suggest that in England and Wales, whilst there were some 150,000 elderly people bedridden or severely physically handicapped in institutions of all sorts, there were some 450,000 elderly people bedridden or severely handicapped living in private households. This latter survey did not involve any medical assessment. Apart from demonstrating conclusively the enormous part played by the community in caring for disabled elderly, one inevitably asks the question "What were the medical causes of disability and in how many could the dependency be reduced or removed?"

How, then, can this situation be resolved? Clearly a first and vital step is a general realisation of the nature and extent of the problem. Within the hospital service the necessary changes are now fairly well defined and accepted by the Ministry of Health even though they have yet to be implemented here in Bristol. The basic hospital geriatric department consists of an acute assessment or diagnostic unit sited within a general hospital; this situation enables those patients admitted directly from the community or through casualty departments to have ready access if necessary to investigations, surgery, or intensive care. This arrangement also favours interaction between the geriatric physician and his colleagues.

Patients posing problems of a physical, mental or social nature, the assessment and resolution of which may require a long time, are transferred to wards where simple physical rehabilitation methods are applied and careful assessment of their functional capacity and mental state is made. Such work involves close liaison between nurse, occupational therapist, physiotherapist social worker, doctor and relatives. Often it is necessary to liaise with the general practitioner, health visitor, welfare officer and district nurse. When doubt exists even after careful assessment about the patient's capacity to function independently, an assessment of the patient in his own home is made before discharge. This process of assessment and rehabilitation is a complicated, difficult, and very time-consuming procedure, having as its objective the happy resettlement of the individual into the community. Provided that the community situation has not deteriorated to the point of rejection before the patient's admission to hospital, such an approach gains the enthusiastic support of the patient, his relatives and friends.

The majority of elderly patients suffer progressive disability and it is important to appreciate that their satisfactory maintenance in the community requires regular and constructive surveillance. Re-admission to hospital for further periods of rehabilitation, or attendance at "day hospitals" may be necessary to maintain a satisfactory balance. It is vital to be able to effect easy interchange between community and hospital. By providing a hospital bed at regular intervals, many otherwise heavily dependent long-stay patients can be maintained happily in their own homes. Help of this sort, given before overstressing induces rejection, is of undoubted value. That any patient should be rejected by a friend or relative who starts to care for him in love and affection and ends by hating him is a terrible indictment of our services in general practice, local authority, and hospital. Intermittent admission permits one long-stay bed to be used by two, three, or even four patients.

Current research suggests that residential homes as we know them now will disappear and be replaced by special housing with or without warden control. It would seem logical for the hospital service to take over existing residential homes for the care of long-term patients who do not require much skilled nursing but who are not fit enough to manage within a sheltered housing scheme. It is obvious that much more specialised housing for the elderly needs to be built, and there is also a great need for day centres to provide opportunities for social intercourse, meals, activities, and bathing.

In all this, however, it seems to me that the general practitioner is the key figure. It is established beyond doubt that the unmet needs for general practitioner care is high amongst old people and the main reason for this is that old people do not report their disabilities until they are advanced. Thus a general practitioner service based on the self-reporting of illness is severely handicapped in meeting the needs of old people.

Several schemes have been proposed in order to facilitate the early detection of disease in the elderly population. Boucher (1967) reported that sixty-seven clinics for old pople had been set up by local authorities with the agreement of the Ministry of Health. But it has been estimated that even if each of the one hundred and eighty local authorities in England and Wales set up three screening clinics they would only see approximately three per cent of the elderly population each year. The examination of those elderly people would occupy the full time of over five hundred medical officers of health. Furthermore, it is likely that those who attend the clinics are not the old people who would most benefit from routine examination.

Recently Williamson (1966) has demonstrated the value of attaching health visitors to group practices to aid screening of elderly people. He has shown that these visitors are efficient at carrying out screening on behalf of the general practitioner, and are skilful in detecting the majority of psychiatric and physical disabilities in old people. Groups of old people who should be visited because they are specially at risk are the recently bereaved, those living alone, those with impaired sight and hearing who are likely to become socially isolated, those with mental disorders, those with locomotor disabilities, the over seventy-fives, and old people who have not been seen by their general practitioners for six months. Exton-Smith and Millard (1968) have pointed out that the idea of surveillance of elderly people at special risk is gaining ground, and suggests that in future the initial screening in the field will be carried out by health visitors and general practitioners and that further investigation will be carried out either in general practice, or in consultative health clinics already established, or in geriatric out-patient clinics.

As I see it, the geriatric problem is a dynamic one and can be best resolved by a dynamic approach, in which general practitioners, local authorities and hospital each have vital complementary roles. Lest there be anyone still in doubt about the need for attention and concern in this field, let me conclude with some words written by Sir Peter Medawar in 1945. "The moral is that the problem of doing something about old age becomes progressively more urgent. Something must be done if it is not to be said that killing people painlessly at the age of seventy is after all a real kindness. Those who argue that our concern is with the preservation of life in infancy and youth so that pediatrics must forever take precedence over what people are beginning to call gerontology fail to realise that the outcome of pediatrics is to preserve the young for an old age that is grudged them. There is no sense in that sort of discrimination."

REFERENCES

Amulree, R.Hon. Lord (1968). Recent Developments in Geriatrics. Abstracts of World Medicine, **42,** 333.

Boucher, C. A. (1957). Survey of Services available to the chronic sick 1954-55. Reports on Public Health and Medical Subjects No. 98. London H.M.S.O.

Boucher, C. A. (1967). 'Meeting the Need' in 'Promoting Health in Middle Life' Pre-retirement Association.

Exton-Smith, A. N. and Millard, P. H. (1968). Early Detection of Disease in the Elderly. Journal of the Medical Women's Federation, 204.

Hobson, W. and Pemberton, J. (1955). 'Health of the Elderly at Home' Butterworth, London.

Medawar, P. B. (1945). The Modern Quarterly. **2**, 30.

Sheldon, J. H. (1948). 'The Social Medicine of Old Age', Oxford University Press, London.

Thomson, A. P. (1949). Brit. Med. J., *ii*, 243, 300.

Townsend, P. and Wedderburn, D. (1965). The Aged in the Welfare State Bell, London.

Wedderburn, D. (1966). Old People's Welfare Council, Scarborough, April 1966.

Williamson, J., Lowther, C. P. and Gray, S. (1966). Geront. clin. Basel, **8**, 36[illegible]

II

### EPIDEMIOLOGICAL ASPECTS OF GERIATRICS

BY

DR. A. W. MACARA

Society is obsessed with "age". Everybody is typed, however irrelevantly, by chronological age, as though the passage of time was the critical test of function. Historical chance and administrative convenience dictate that the Englishman's 65th birthday, and anomalously his sister's 60th, labels them "elderly". Old age is 65. The gold watch, the embarrassing speeches, the "well done, thou good and faithful servant". Retire decently, old man, to your rocking chair and carpet slippers. The ritual write-off. Thus, the community, ambivalently well intentioned—"you have earned your rest", "one must make way for youth"—patronizes the elderly into second-class citizenship. Made to expect little, man asks for less and neglects to exercise even his remaining rights.

Sadly, doctors are not immune from infection by this insidious attitude of mind. Do we not tend to regard a degree of illness and incapacity as inevitable in old age? May it be realization of *our* attitude which so often makes the elderly patient endure in silence those minor disabilities which may herald chronic incapacity?

We may enter one plea in our defence. Fifty years ago, 29% of all deaths in males aged 75 and over were blandly assigned to the rubric called "old age". Today this category, now known as "senility", claims under 2% of total deaths in this group.[5] Such a change in certification practice reveals some improvement in attitudes.

However much we may deplore it, the age of 65 has profound implications; not least because the baseline information about geriatrics is available only in terms of the arbitrary age group 65 and over (and occasionally 60 and over for females). We hear much about the "ageing" of the population—the increasing proportion of the population which is aged 65 and over in this country and elsewhere. What are the facts?

Figure 1 shows the percentage of the total population aged 65 and over in various countries chosen to allow comparison between parts of the world in different stages of demographic development.[3] In 1966, according to the "sample census" figures of that year, there were six million people aged 65 and over in England and Wales, constituting 12% of the population,[9] compared with $1\frac{1}{2}$ million or 5% of the population in 1901. This fourfold rise in the number of the aged is much greater than the rise in the number of people in the other age groups, which explains the increased proportion of the total population which is elderly. This proportion is increasing all the time, and we must remember that this elderly 12% of the population is not equally distributed throughout the country. The figure for Bristol is nearer 14% and in Clifton 17%. Eastbourne tops the league with 27.5%.

By way of contrast, the number of children aged 0-14 in England and Wales today is almost exactly the same as in 1911, despite an increase in the total size of the population of almost 50%. This is due to a reduction in the fertility rate shortly after the turn of the century, and this phenomenon is mainly responsible for the increased proportion of the elderly in the population at the present time. Until now, the dramatic reduction in death rates at younger ages has made only a secondary contribution to the "ageing" of the population. Fertility rates have now become relatively stable and their full effect on the ageing of the population has probably now been experienced. The consequences of the reduction of mortality are yet to be fully felt, and the inevitable result of improved survival rates among children and young adults over the last 30 years will be a permanent and continuing increase in the number of the elderly in the community. So the triumphs against poverty and disease enable a rising proportion of people to realize the biblical life span and more.

The expectation of life at birth has increased since 1841 from 40 to over 68 years for males, and from 42 to over 75 years for females. But what of the expectation of survival at the threshold of old age? The expectation of life for the 65 year old man has increased by only about 2 years in the last 7 decades—from 10 to 12 years.[5] So there has been as yet little progress in increasing the total life span. There has been correspondingly little progress in improving the health of the elderly. Space does not permit details to be given, but it is clear that the elderly have not shared in the dramatic improvement in mortality and morbidity experience enjoyed in recent years by the young age groups. Whatever the reasons for this, we are promised a continuing increase in the number of old people, and common humanity dictates that we do our best to improve their prospects. Tantalizingly, such improvements will increase the proportion of the elderly and especially of the very elderly in the population still further. The Registrar-general obviously expects this. His population projections indicate a 17% increase in total population by 1991. Although the increase in the 65-74 age group is expected to be only 6.8%, in the age group 75 and over—the very elderly—it is put at 35%.[10] But surely we should be happier if people *did* live longer, so long as they were fitter, more active and less dependent. As Professor Neale has put it, "Oh to die young, but late".

TABLE I

*Population aged* 65 *and over by sex, age and widow(er) status,* 1966, *England and Wales.*

| | | Total | Widowed | % Widowed |
|---|---|---|---|---|
| Males: | 65—74 | 1,530,660 | 214,880 | 14 |
| | 75+ | 698,410 | 261,390 | 37 |
| | Total | 2,229,070 | 476,270 | 21 |
| Females: | 65—74 | 2,225,950 | 888,660 | 40 |
| | 75+ | 1,400,700 | 916,370 | 65 |
| | Total | 3,626,650 | 1,805,030 | 50 |
| Persons: | 65—74 | 3,756,610 | 1,103,540 | 29 |
| | 75+ | 2,099,110 | 1,177,760 | 56 |
| | Total | 5,855,720 | 2,281,300 | 39 |

FIGURE 1. *Percentage of persons aged 65 and above from* 1900 *onwards in different countries.*

What can be done about all this? We need to know more about the needs of the elderly before devising means to meet these needs. Table 1 analyses the population aged 65 and over as at the 1966 sample census. Note that at age 75 and over, the ratio of females to males is 2:1, demonstrating the biological superiority of the " weaker sex " of whom 2/3rds aged 75 and over are widows, i.e. 1 million in England and Wales. Including women aged 65-74, there are 2 million elderly widows compared with only 0.5 million elderly widowers. On the other hand, half of all the elderly are married.

Where and with whom do the elderly live? Table II summarises material from Townsend and Wedderburn's study " The Aged in the Welfare State ".[7] So much attention is focused upon the elderly who live in institutions or who are neglected by their relatives that it is worth noting that 94% live at home. And of those, 2/3rds live with or near children or relatives. Moreover, as Tunstall showed, 78% of the elderly living in private households do not live alone.[8] On the other hand, 2 million *are* without a relative living with them or nearby and 1.4 million live completely alone. Of these 1.4 million, 300,000 are significantly incapacitated. Put another way, there are as many incapacitated elderly people living alone at home as there are in all the institutions put together. They constitute probably the greatest problem of all. Nevertheless, very few old people who have children are isolated from them, and many who live alone do so from choice. There is no evidence to support the damaging myth that the elderly are neglected by their families despite difficulties created by inadequate housing and the increased mobility of the younger members of families. On the contrary, it is suggested that but for the care given to sick and infirm elderly people by the family, often at considerable sacrifice, the burden on the Health and Welfare Services would be three to five times greater than it is.[6]

TABLE II

*Place of residence and proximity of relatives of those aged 65 and over, Great Britain, 1965.*

| | Number | | % | |
|---|---|---|---|---|
| Private households | | | | |
| a. Children living at home ... | 1,942,000 | | 30 | |
| b. Other relatives living at home | 592,000 | | 9 | |
| c. No relatives at home, children near ... ... ... | 953,000 | | 15 | |
| d. No relatives at home, other relatives living near ... ... | 666,000 | | 10 | |
| e. No relatives at home or living near ... ... ... | 1,955,000 | | 30 | |
| | | 6,108,000 | | 94 |
| Residential homes ... ... ... | | 110,000 | | 2 |
| Psychiatric hospitals and nursing homes | | 63,000 | | 1 |
| Other hospitals ... ... ... ... | | 121,000 | | 2 |
| Hotels, etc. ... ... ... ... | | 100,000 | | 1 |
| Total ... ... ... ... ... | | 6,502,000 | | 100 |

How many of the elderly are gainfully employed? The figures in Table III demonstrate that old age and retirement are not invariably synonymous. Encouraging though this may be, Benjamin reminds us that in 1921 when no widespread compulsory retirement system operated, 89% of men aged 65-69, and 27% of men aged 75 and over were employed.[1] Many of these no doubt worked from necessity rather than desire, but the discrepancy between these figures and the present day ones do suggest that greater flexibility in retirement age could be advantageous.

TABLE III

*Employment status of those aged* 65 *and over, Great Britain,* 1961.

(Source: 1961 Census Report, Summary Tables)

| | Economically Active | | Part-time Workers | |
|---|---|---|---|---|
| | No. | % of total | No. | % of economically active |
| Men: | | | | |
| 65—69 ... ... | 361,970 | 40 | 57,580 | 16 |
| 70—74 ... ... | 138,810 | 21 | 39,050 | 28 |
| 75+ ... ... | 69,540 | 10 | 17,910 | 30 |
| Total ... ... | 570,320 | 25 | 114,540 | 21 |
| Women: | | | | |
| 65—69 ... ... | 130,060 | 10 | 59,460 | 46 |
| 70—74 ... ... | 48,580 | 5 | 19,500 | 40 |
| 75+ ... ... | 23,820 | 2 | 5,900 | 25 |
| Total ... ... | 202,460 | 5 | 84,860 | 42 |

How badly off are the elderly financially? Few of them have substantial savings or assets, the very elderly and the single or widowed woman being particularly poor. On the other hand the large majority of the aged receive state pensions and benefits, but as many as 11% have been estimated not to claim their full entitlement; and even when all available benefits are applied, the income levels are less than half those of the general population.[7]

What demands do the elderly make on the Medical Care Services? A few facts are revealing. The elderly 12% of the population accounts for 34% of the expenditure on hospital services and occupies a similar and steadily increasing percentage of hospital beds.[5] But they make remarkably low use of hospital out-patient facilities. Our 12% also account for 24% of expenditure on the General Medical Services and between 18 and 21% of all consultations with G.P.s.[4] They also absorb 21% and 19% by value of pharmaceutical and ophthalmic services respectively.[2] One may speculate about the significance of these disproportionate demands upon the "cure" services. Do these figures give the lie to the argument that old people, resigned to minor and even major ailments as inevitable concomitants of old age, fail to surrender in time to the doctor? Or conversely, is it because of delay and inadequacy in diagnosing and treating their disabilities that they eventually absorb so much attention for disabilities which could have been prevented?

What of the other medical social services? Voluntary bodies with Local Authority support, such as Bristol Old People's Welfare and the Women's Royal Voluntary Service make a major contribution to the welfare of the elderly, but of course the main brunt falls upon the statutory services and the elderly make heavy demands upon the Local Health Authority Services, accounting for 27% of their expenditure under the National Health Service Acts.[5] The elderly absorb about 70% of the work of district nurses and practically monopolize the Home Help Service. Despite these demands there are many unmet needs. Indeed the need for any service such as domiciliary chiropody is rarely fully recognised until it is introduced. People can use only what is there. Table IV illustrates the extent to which various needs remain unmet.

TABLE IV

*Uses and unmet needs of those aged 65 and over, living in private households, 1966, Great Britain.*

(Source: Derived from 'The Aged in the Welfare State' by P. Townsend and D. Wedderburn).

| Service | Number | % of all aged 65 and over living in private households |
|---|---|---|
| *Home Helps* | | |
| 1. Receiving ... ... ... ... | 268,000 | 4 |
| 2. Feeling a need for but not receiving | 348,000 | 6 |
| 3. Not feeling a need but having difficulty and no help ... ... ... | 281,000 | 5 |
| *Meals* | | |
| 1. Receiving cooked meal from mobile service ... ... ... ... | 69,000 | 1 |
| 2. Expressing a wish for hot meal from mobile service but not receiving one ... ... ... ... | 361,000 | 6 |
| *Chiropody* | | |
| 1. Having chiropody, public or voluntary | 446,000 | 7 |
| 2. Having chiropody privately paid ... | 690,000 | 11 |
| 3. No chiropody but feeling a need for some ... ... ... ... | 703,000 | 13 |

What of the fear that provision of more adequate services will increase dependence upon society, and discourage the self-help which is probably the greatest need of all? First we need to do more work to seek out the proudly independent elderly person who does not seek help until it is too late to preserve continued independence. Secondly, we might reconsider our priorities and build up those services which promote and preserve self-help and independence in old age.

We are now familiar with the concept of the " Clinical Iceberg " phenomenon. How does it affect the elderly? How much undiagnosed disability is there in the ageing body? Table V summarises material from Last's work. It shows how much trouble late in life is already predictable at an earlier age. The consequences of failing to detect such trouble in good time are illustrated in Table VI.[11] This study by the Royal College of Physicians in Edinburgh in 1962 in which they investigated Sheldon's allegation[12] of a high prevalence of significant minor ill health in the elderly, speaks for itself.

TABLE V

*" The Clinical Iceberg ". England and Wales.* 1962.
(Source: J. M. Last, Lancet, 1963, II, 28.)

| Disorder | | Number of recognised sufferers 1,000s | Number of unrecognised cases 1,000s |
|---|---|---|---|
| Hypertension ... ... | Males 45+ | 170 | 450 |
| | Females 45+ | 500 | 2,220 |
| Urinary infections ... | Females 15+ | 420 | 410 |
| Glaucoma ... ... | Aged 45+ | 60 | 280 |
| Epilepsy ... ... | | 160 | 120 |
| Rheumatoid arthritis ... | Aged 15+ | 230 | 290 |
| Psychiatric disorders ... | Males 15+ | 560 | 640 |
| | Females 15+ | 1,290 | 830 |
| Diabetes mellitus ... | | 290 | 310 |
| Bronchitis ... ... | Males 45-64 | 500 | 480 |
| | Females 45-64 | 390 | 110 |

TABLE VI

*Recognised and unrecognised disabilities in those over* 65 *years, Edinburgh,* 1962.
(Source: Royal College of Physicians, Edinburgh, ' The Care of the Elderly in Scotland ', 1963).

| Diagnosis | Percentage known to be suffering by their general practitioner | Total percentage with disabilities (known and unknown) |
|---|---|---|
| Disability of the feet ... ... ... | 5.5 | 43.0 |
| Disability of the locomotor system (excluding feet) ... ... ... ... | 21.0 | 37.0 |
| Dementia ... ... ... ... ... | 3.5 | 27.5 |
| Disease of urinary tract ... ... ... | 2.5 | 12.0 |
| Anæmic ... ... ... ... ... | 1.0 | 8.0 |

The implications of this are legion. I single out just two. Do wo not need screening services for the elderly on a national scale such as those at Rutherglen and our own St. George Health Centre, to establish a baseline of information about physical, mental and social health? Perhaps such a service should anticipate retirement rather than accompany it. More important even than this, we should do everything we can to encourage activity of body, mind, and spirit. Above all, we must see the elderly in physiological rather than pathological terms.

REFERENCES

1. Benjamin, B., 1957. "Demographic Aspects of Ageing", in "The Biology of Ageing", Inst. of Biology.

2. British Medical Index, 1966. Intercontinental Medical Statistics Ltd.

3. Hobson, W., 1965, in "Theory and Practice of Public Health" 2nd Ed. p. 318, O.U.P.

4. Logan, W. P. D., Cushion, A. A., 1958, Morbidity Statistics from General Practice, Vol. 1, General Register Office, H.M.S.O.

5. Office of Health Economies, 1968, "Old Age".

6. Townsend, P., 1957, "The Family Life of Old People", Routledge and Kegan Paul.

7. Townsend, P., Wedderburn, D., 1965, "The Aged in the Welfare State", G. Bell and Sons.

8. Tunstall, J., 1966, "Old and Alone", A Sociological Study of Old People, Routledge and Kegan Paul.

9. Registrar-General's Statistical Review of England and Wales, 1965, Pt. II.

10. Report of the Committee on Local Authority and Allied Personal Social Services ("Seebohm" Report) Cmnd. 3703, 1968, p. 91, H.M.S.O.

11. Royal College of Physicians of Edinburgh, 1963, "The Care of the Elderly in Scotland".

12. Sheldon, J. H., 1948. "The Social Medicine of Old Age", O.U.P. for the Nuffield Foundation.

### III

### A GENERAL PRACTITIONER'S VIEW

BY

DR. P. S. SINCLAIR

Nearly two million of our population are 75 and over, and one and a half million live alone. Many have no human contact with the outside world. These and the recently bereaved are the groups that are at the greatest risk, and they are particularly vulnerable. They are mostly not in hospitals; in spite of advances in geriatrics and our ability to diagnose those in need of hospital-isation, there is no room for them. Many of my colleagues know from bitter experience how difficult it is to get a patient over 75 admitted. Sometimes these patients will die, lonely and not cared for, whilst a bed is being found. More than 95% (over six million) live in their own homes and are looked after (medically) by their general practitioners. This gives the G.P.a great chance to observe, and if necessary treat them. It puts a tremendous respons-ibility and duty upon all of us, and gives us an opportunity which only a few people in the community share with us.

The fact that people over 65 should be termed old is the biggest blow that society has inflicted upon humanity. It helps to destroy the morale and self-respect of the senior citizen.

I started to be interested in the social welfare of the elderly over two years ago, when I failed to get a " sitter-in " for a patient who had partially recovered from a subarachnoid hæmmorhage (Plate VI). She had a hemi-plegia, was aphasic, and could only dress herself with the aid of her husband. Without additional help the husband would have had to give up his work—and he was 56. None of the existing statutory and voluntary bodies was able to assist. I then thought that the problem for similar cases could be solved if we could have a panel of elderly people who could sit in at *any* time. I talked to two old age clubs explaining my proposition and asking for volunteers saying that the fit should help the not so fit. I finished by saying " You may be the next one that needs help '.

The result was the formation of SHE 7. " SHE " stands for Self Help Enterprise; 7 is the postal district of Bristol. The title proved to be a fortunate choice; it gave us valuable publicity. " SHE and her vital statistics " was the first headline. The following morning the *News of the World* telephoned and and sent a reporter. After this many reporters followed. I learned a lot from these interviews, mainly what *not* to say. The response to my appeal for helpers was overwhelming. There were suddenly 25 willing helpers and only two patients to be helped. I had to look for deserving cases, to keep alive the enthusiasm of the volunteers. Meanwhile, I kept them busy by starting a meals-on-wheels service in our area, which for some reason or other did not then exist.

An example of how the home visiting scheme shows its usefulness is that of a man of 83 who lived with his daughter. Whenever the daughter went out, the father started wandering about, and he was usually returned by the Police in the early hours. We supplied a day sitter within twenty-four hours

PLATE VI.—*SHE 7's first visiting case, a hemiplegic lady (with her husband aged 56) and our visitor.*

PLATE VII.—*This helper, aged 78, has offered to be the foreman of SHE 7's workshop.*

From that day onwards the daughter could go shopping, and the household ran smoothly until father was admitted to Manor Park Hospital four weeks later.

Of course, we encountered difficulties; the elderly who needed help would not accept it. So I had to change my tactics, asking those whom I thought were in need of support if they would do *me* a personal favour—would they find out for me whether a future visitor was suitable for the job? I explained that the trainee would come once a week for six weeks; after that time, I said, " I will ask *you* for *your* opinion, and your decision will be final ". When I put it this way, nine out of ten patients accepted, and none wanted to part with the visitor after six weeks. I learned by this trick that if we want to help the lonely and housebound we must find ways and means to be accepted by them.

SHE 7 has successfully started several pilot schemes which I hope will provide a pattern for other organisations. We train home visitors over 60 for their job by asking them to attend lectures given at Filton Technical College. It is not easy to persuade the eldely to go back to school, and to make it more acceptable we have introduced some visual aid. After the course of lectures, the Drama Department of the Technical College produced a sketch to " re-cap." what had been learned.

It is generally recognized that some form of part-time employment after retirement keeps the elderly not only healthier, but happier. It prevents psychological trauma to a person who has not developed a worth-while hobby. Women of this age group occupy themselves mainly with housework and social activities; this is one of the many reasons why at the age of 65 there are twice as many women alive as men. Another is probably the fact that no self-respecting husband can stand his wife's nagging for more than 40-odd years. He prefers to die. SHE 7 dealt with women and how to keep them usefully occupied, thereby helping themselves and the housebound patient they visit.

The next pilot scheme that we are about to launch is intended for *men* over 65. We propose to start our part-time employment scheme in leather-work (mainly repairs), light engineering, and crafts (Plate VII). Now, after one year's struggle, the Gloucestershire County Council has agreed to let to us two large buildings that were practically unused. I am glad to say that, in the end, even County Councils do wake up to their responsibilities towards the elderly. Similar workshops were started nearly 20 years ago by Rubery Owen (Staffs.), and were followed by Rolls Royce and several other firms. From this country the workshop idea spread, notably to Holland, Sweden, Germany, and the U.S.A. About 60 workshops exist in Great Britain, some of them run successfully, but many have a loss of £1,000 a year. We believe that our SHE 7 workshop will be viable, that we shall get the support of the community, and that we shall make a profit.

In an ever increasing geriatric population we need all available people and every possible help to reach our ultimate aim of making retirement not a burden, but a pleasure to look forward to. To get a scheme of this sort under way, the general practitioner must not only support it, he must take the lead.

## Figures and Tables

**Figure 1. f1:**
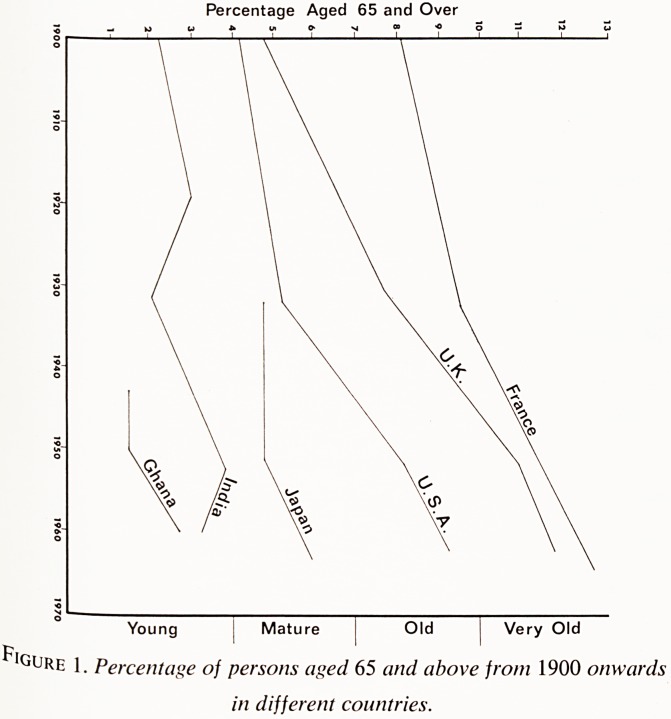


**Plate VI. f2:**
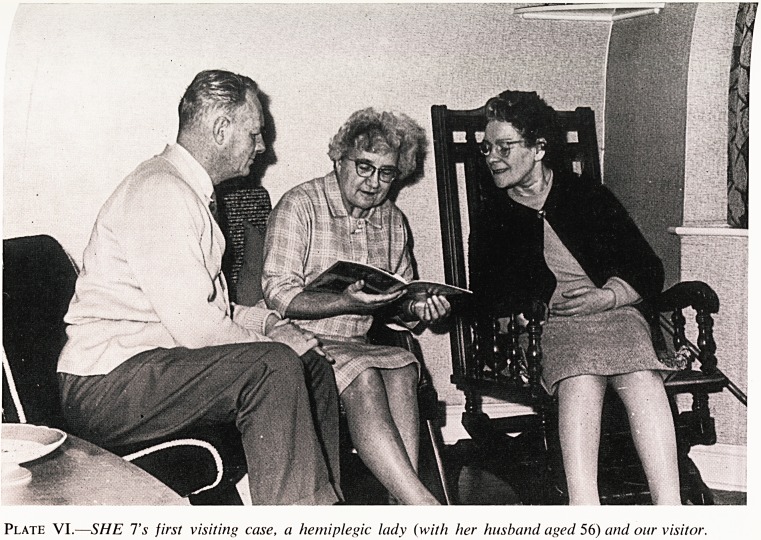


**Plate VII. f3:**